# Genomic analysis of ERVWE2 locus in patients with multiple sclerosis: absence of genetic association but potential role of human endogenous retrovirus type W elements in molecular mimicry with myelin antigen

**DOI:** 10.3389/fmicb.2013.00172

**Published:** 2013-06-26

**Authors:** Guilherme S. do Olival, Thiago S. Faria, Luiz H. S. Nali, Augusto C. P. de Oliveira, Jorge Casseb, Jose E. Vidal, Vitor B. Cavenaghi, Charles P. Tilbery, Lenira Moraes, Maria C. S. Fink, Laura M. Sumita, Hervé Perron, Camila M. Romano

**Affiliations:** ^1^Departamento de Neurologia, Irmandade da Santa Casa de Misericórdia de São PauloSão Paulo, Brazil; ^2^Departamento de Moléstias Infecciosas e Parasitárias – (LIMHC), Instituto de Medicina Tropical de São Paulo e Faculdade de Medicina, Universidade de São PauloSão Paulo, Brazil; ^3^Departamento de Neurologia, Instituto de Infectologia Emilio RibasSão Paulo, Brazil; ^4^Laboratório de Imunodeficiências e Dermatologia, Instituto de Medicina Tropical de São Paulo e Faculdade de Medicina, Universidade de São PauloSão Paulo, Brazil; ^5^Departamento de Neurologia, Hospital das Clínicas da Faculdade de Medicina, Universidade de São PauloSão Paulo, Brazil; ^6^Geneuro, Plan Les OuatesGeneva, Switzerland

**Keywords:** multiple sclerosis, immunopathogeny, endogenous retroviruses, ERVWE2, genetic association, MOG

## Abstract

Human endogenous retroviruses (HERVs) arise from ancient infections of the host germline cells by exogenous retroviruses, constituting 8% of the human genome. Elevated level of envelope transcripts from HERVs-W has been detected in CSF, plasma and brain tissues from patients with Multiple Sclerosis (MS), most of them from Xq22.3, 15q21.3, and 6q21 chromosomes. However, since the locus Xq22.3 (ERVWE2) lack the 5′ LTR promoter and the putative protein should be truncated due to a stop codon, we investigated the ERVWE2 genomic loci from 84 individuals, including MS patients with active HERV-W expression detected in PBMC. In addition, an automated search for promoter sequences in 20 kb nearby region of ERVWE2 reference sequence was performed. Several putative binding sites for cellular cofactors and enhancers were found, suggesting that transcription may occur via alternative promoters. However, ERVWE2 DNA sequencing of MS and healthy individuals revealed that all of them harbor a stop codon at site 39, undermining the expression of a full-length protein. Finally, since plaque formation in central nervous system (CNS) of MS patients is attributed to immunological mechanisms triggered by autoimmune attack against myelin, we also investigated the level of similarity between envelope protein and myelin oligodendrocyte glycoprotein (MOG). Comparison of the MOG to the envelope identified five retroviral regions similar to the Ig-like domain of MOG. Interestingly, one of them includes T and B cell epitopes, capable to induce T effector functions and circulating Abs in rats. In sum, although no DNA substitutions that would link ERVWE2 to the MS pathogeny was found, the similarity between the envelope protein to MOG extends the idea that ERVEW2 may be involved on the immunopathogenesis of MS, maybe facilitating the MOG recognizing by the immune system. Although awaiting experimental evidences, the data presented here may expand the scope of the endogenous retroviruses involvement on MS pathogenesis.

## Introduction

Multiple Sclerosis (MS) is an immune-mediated disease of the central nervous system (CNS), but precise immunologic mechanisms involved in the induction and chronic course of MS are still poorly understood. Epidemiological studies have shown both environmental and genetic factors to display an association with this disease. Genetic predisposition includes some familiar inherited risk, the association to the HLA class II DRB1^*^1501 and DRB5^*^0101 alleles in Caucasians (Gregersen et al., 2006) and an abnormal autoimmune reaction against the prominent CNS myelin antigens such as the myelin oligodendrocyte glycoprotein (MOG). MOG is a transmembrane protein expressed in oligodendrocytes with extracellular Ig domain that works as an important CNS-specific autoantigen. Nevertheless, despite evidence of humoral and T cell-mediated responses to myelin basic protein (MBP) and MOG (Bernard and de Rosbo, 1991), unambiguous relationship between myelin reactivity and disease has yet to be demonstrated.

An infectious etiology of MS has been also postulated, and over several years a number of different viruses have been suggested to trigger immunopathology. One mechanism by which virus infection may lead to autoimmune reaction is via expression of superantigens. Superantigens are molecules that induce activation and proliferation of nonspecific T cells, often leading to a chronic inflammatory response. In addition to the superantigens, molecular mimicry-activation of autoreactive T cells by viral epitopes that are shared or cross-reactive with self-antigens could also lead to an autoimmune response (Colmegna and Garry, 2006).

Increased antibody titers were detected for Epstein-Barr, Varicella-Zoster, and Rubella Viruses, whereas specific proteins of human endogenous retrovirus (HERV), particularly from HERV-W family were evidenced in blood cells and brain lesions of MS patients (Antony et al., 2004a; Mameli et al., 2007; Owens et al., 2011; Perron et al., 2012). Also, EBV and HERV-W-associated superantigens where already hypothesized to contribute to MS pathogeny (Tai et al., 2008; Antony et al., 2011).

HERVs arise from ancient infections of the host germline cells by exogenous retroviruses and today, constitute about 8% of the whole human genome (Lander et al., 2001). A number of studies have found increased expression of HERV genes from different families in individuals presenting autoimmune diseases (Bannert and Kurth, 2004; Ehlhardt et al., 2006; Schulz et al., 2006; Perron and Lang, 2010).

Elevated RNA levels of MRSV (Multiple Sclerosis associated Retrovirus), a prototypic element defining a subtype from HERV-W family, have been frequently detected in CSF, plasma and brain tissues from patients with MS but rarely in healthy control individuals. (Perron et al., 1997, 2012; Garson et al., 1998; Antony et al., 2004b; Dolei and Perron, 2009). MSRV sequences were found to be associated with extracellular retroviral particles in MS, but may also originate or be co-activated from different proviral copies (Komurian-Pradel et al., 1999), besides also diverge in quantity and identity between individuals (Mameli et al., 2009; Perron et al., 2012). Recently, it was described that many HERV-W envelope transcripts from MS patients derived from Xq22.3, 15q21.3, and 6q21 (Laufer et al., 2009). Although the genomic Xq22.3 MRSV/env (ERVWE2) has no 5′ LTR promoter and is truncated due to a stop codon (TGA) at position 39, the authors found several transcripts associated to this locus, but harboring a tryptophan (TGG) at site 39 instead. These findings open the possibility for an envelope protein encoded by an HERV-W element of the MSRV subtype (Mameli et al., 2009) plays a role in MS.

Thus, given the clear association of HERV-W elements to MS, the aim of this study was to investigate the putative involvement of Xq22.3 HERV-Wenv locus (ERVWE2) in MS pathogeny combining molecular and bioinformatics tools. This report also presents *in silico* evidences that a retroviral envelope expression could trigger the adaptive immune response against brain antigens. Although awaiting formal and conclusive evidence, our data may have important implications for redefining the breadth of the ERVWE2 involvement on autoimmunity. Furthermore, since the contribution of the endogenous retroviruses to human diseases remains largely unexplored, dissemination of knowledge at this level is worth in this domain.

## Patients and methods

### Samples

The current project was conducted after Hospital das Clínicas - University of Sao Paulo's Institutional Review Board (CAPPesq) approval, under protocol #0166/11. Written informed consent was obtained from all participants.

Total blood was obtained from 47 patients with MS diagnosed according to McDonald criteria, revised by Polman et al. (2011). We also included 37 healthy individuals with no familiar history of MS. The cohort investigated included patients classified as relapse—remitting, primary, and secondary progressive MS.

### Reverse transcription and HERV-W envelope real time detection

HERV-W envelope expression was investigated in a subset of samples (10 of 47 MS patients) that had peripheral blood mononuclear cells (PBMC) stored. As a control, envelope transcripts were investigated in PBMC from the same number of healthy individuals (*n* = 10). Total RNA was isolated from cells using Trizol LSReagent® (Invitrogen Corporation, Carlsbad, CA). Samples were pre-treated twice with DNAse to eliminate traces of genomic DNA as a source of putative HERV amplified sequences. Reverse transcriptase (RT) was performed with High-Capacity cDNA Reverse Transcription Kit (Applied Biosystems) and amplifications without RT enzyme were done in parallel to check for remaining DNA on samples. HERV-W envelope primers and RT-qPCR conditions were set according to previous description (Nellåker et al., 2006), 50ng of cDNA was used as input for RT-qPCR and the relative expression was normalized according to Beta-actin expression through Human ACTB (Beta Actin) Endogenous Control kit (Applied Biosystems).

### ERVWE2 amplification and sequencing

After genomic DNA extraction using QIAamp DNA Blood Mini kit (Qiagen), a 896 base pairs (bp) of envelope region corresponding to nucleotides 21231 to 22127 of human chromosome Xq22.3 MRSV locus (ERVWE2, GenBank ID AL390039.10) was amplified by PCR using following specific primers: HW_MSchX_F-CTGTTGGACTTACTTCACCCA and HW_ MSchX _R- TGAAGAACGTATCCAGCCTACA. The thermal profile for amplification was 38 cycles as follows: 94°C for 45 s, 52°C for 1 min, and 72°C for 1 min. PCR products were purified using PEG 20% (polyethylene glycol) and sequenced on an ABI 3100 genetic analyzer (Applied Biosystems, Foster City, CA). The eletropherograms were analyzed and consensus sequences were achieved with CodonCode Aligner v.3.0 (available at http://www.codoncode.com/).

### Sequencing and bioinformatic analysis

Nucleotide sequences obtained from MS and healthy individuals were manually edited and aligned to reference sequences from ERVWE2 DNA and transcripts obtained by Laufer et al. (2009) in SeAl (available at http://tree.bio.ed.ac.uk/software/seal/). The aligned dataset was translated to amino acid and the putative substitution TGA/TGG at codon 39 was visually inspected. In addition to the ERVWE2, DNA sequences from MRSV envelope gene present in other chromosomes were also retrieved using BLAT tool (www.genome.ucsc.edu). Sequences were manually aligned, translated and visually inspected.

Since ERVWE2 lacks the 5′LTR promoter, we performed an automated search for alternative promoters and regulatory regions in 10 kb region up and downstream of the envelope gene in the GenBank reference sequence (AL39009.10, position 10079 to 32008) using Cister (Cis-element Cluster Finder) (Frith et al., 2001) and PromoterScan (http://www-bimas.cit.nih.gov/molbio/proscan/) tools.

Since the HERV-W envelope can work as autoantigen and hypothetically, serve as trigger for autoimmune disease, we also evaluated the level of similarity between HERV-W envelope protein and the different isoforms of MBP and MOG using Blastp tool (ftp://ftp.ncbi.nlm.nih.gov/blast/executables/blast+/LATEST/). Some search parameters were adjusted to increase the probability of detecting significant matches (i.e., Expected threshold = 11 and Gap costs: existence = 10 and extension = 1). Prediction of cleavage sites for human proteases was performed in MOG and HERV-W envelope using PAProc I (http://www.paproc.de/). Finally, NetGene2 (available at: http://www.cbs.dtu.dk/services/NetGene2/output.php) was used to predict putative donor and acceptor splicing sites in the ERVWE2.

## Results

Forty-seven patients with clinically definite MS and 37 healthy individuals were enrolled in the study. Among the patients, 6 were male and 41 female, with mean age of 27 years. Volunteers were classified according to the type of MS as follows: 40 had relapsing-remitting clinical form, 6 had secondary progressive form, and 1 had primary progressive form.

### HERV-W expression detection

As expected, most patients presented HERV-W envelope activity in some level. Nine from ten samples tested presented medium to high levels of HERV-W envelope expression, against very low levels of expression (five individuals presented basal HERV-W activity) or no activity at all (five individuals) in healthy controls, confirming the association between MS and HERV-W activity in our population. Although we did not sequence the cDNAs, the Real-Time results suggest that is some variability among the sequences, as indicated by variations in Tm's of transcripts amplified from distinct patients (ranging from 76.1 to 77.6) (Nellåker et al., 2006).

### ERVWE2 genetic investigation

Since the genomic reference sequence from ERVWE2 is truncated due to a stop codon at position 39, we have searched for genetic changes on the DNA of MS patients that could revert the stop codon and produce a full- length protein. To do this, 896 bp of ERVWE2 from 47 MS patients were amplified, sequenced and compared to the genomic DNA from healthy individuals. We found that all MS patients and healthy individuals included in this study harbor a stop codon at position 39, thus preventing the expression of a full-length Env protein. No significant additional substitution was found in MS patients that differentiate them from healthy individuals. GenBank IDs of sequences generated in this study are JX293193-JX293276.

**Figure 1:**
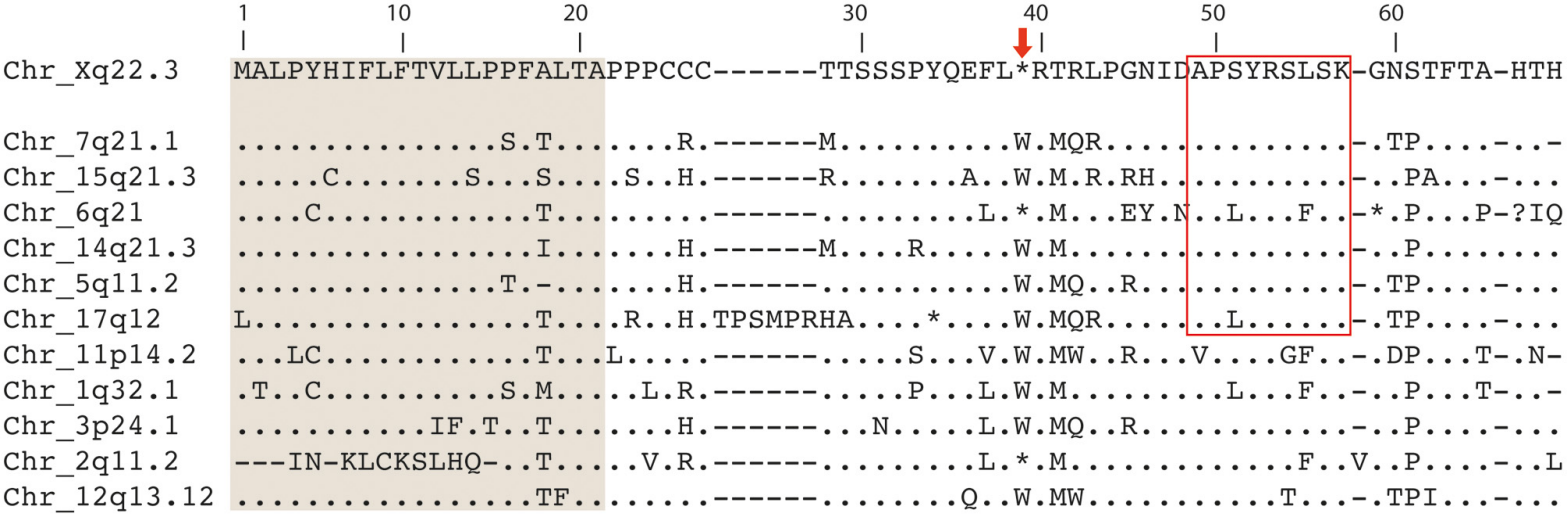
**Multiple alignment of twelve HERV-W envelope proteins located in distinct chromosome loci**. Predicted signal peptides are shaded in gray (Laufer et al., 2009). The thin box evidence the peptide corresponding to coordinates 49–57 found to be similar to MOG epitope in Blastp analysis. The arrow in the top indicates the stop codon at position 39, present in Xq22.3, 6q21, and 2q11.2.

Because of the inability of ERVWE2 to produce a complete ORF in both healthy and MS individuals, we then investigated the genetic integrity of eleven additional HERV-W envelope copies located at different chromosomes available on the GenBank. The amino acid-translated genomic sequence from 6q21 HERV-W and 2q11.2 copies also presented a stop codon at position 39, and 17q12 envelope has a stop codon at position 34 (Figure 1). Altogether, these data suggests that three of the five most active HERV-W loci can only produce N-terminally truncated proteins.

**Figure 2:**
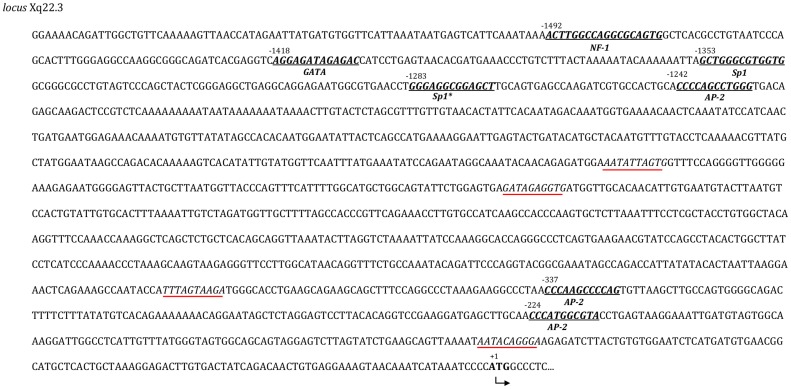
**Transcription factors binding sites (TFBs)**. TFBs and TATA boxes were mapped through the 1580 upstream region before ATG of the Xq22.3 envelope region. The seven putative regions are in bold and italicized. The coordinate position and the name of the putative transcription factor are also specified. Putative TATA boxes are italicized and underlined.

Despite that ERVWE2 can produce only truncated proteins, its expression is indubitable since has been repeatedly detected by many authors. Since ERVWE2 lacks a formal promoter, the automated search for alternative promoter sequences in the vicinity of the ERVWE2 was performed and revealed seven transcription factors binding (TFB) sites with high probability (>0.8) to act as regulatory elements located up to 1.6 kb upstream of the envelope gene (three for AP-2, two for Sp1, one for NF-1, one GATA and four putative TATA boxes) (Figure 2). One of them, the Sp1 at position −1283 bp was identified with high posterior probability in both prediction programs used.

### HERV-W and myelin similarity search

**Figure 3:**
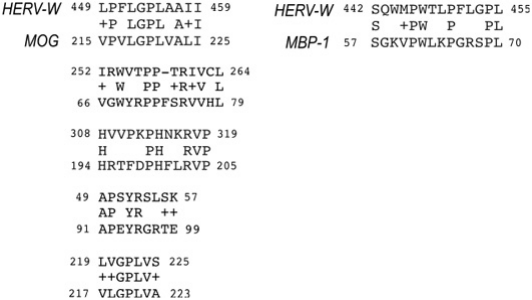
**Epitope regions shared between ERVWE2 envelope and myelin proteins (MOG and MBP)**. The aminoacid alignment was generated by Blastp tool and significant matches are shown. Coordinate positions of each protein are indicated after identification. Plus signals correspond to aminoacids having the same polarity characteristics. The MOG peptides corresponding to coordinates 66–79 and 91–99 are located at extracellular domain of the protein.

Because of the relatively high level of conservation among members of HERV-W envelope genes from subfamily 3, Blastp analysis were performed to compare the complete sequence of HERV-W envelope protein encoded by virion-associated retroviral RNA (MRSV type) (Komurian-Pradel et al., 1999; Perron et al., 2001) to different isoforms of MBP, isoform 1 (EAW66598) and isoform 4 (EAW66598) and MOG (ID CAQ06616). Five retroviral regions were found to have significant similarity to MOG (Expected value = 0.1) (Figure 3), two of them are within the Ig-like domain and were predicted as extracellular domain in Protein Databank (PDB, at http://www.rcsb.org/pdb/protein/Q63345). Interestingly, one of these extracellular regions (MOG_66–79_) includes T and B cell epitopes, which are capable to induce both T effectors functions and circulating Abs in rats (Ichikawa et al., 1996).

The second region within the extracellular domain (MOG_91–99_) shares six aminoacids to the retroviral envelope (four identical and two with the same physical-chemical properties) and was conserved among all loci previously associated to transcripts in MS patients, as illustrated in Figures 1, 3 (Laufer et al., 2009).

A single match was found between the MBP and retroviral envelope that overlaps the region shared between HERV and MOG in a helical domain (MOG_215–225_). Blastp of MBP isoform 4 and retroviral envelope did not result in significant matches.

Finally, since a cross-reactive response against MOG would be induced through the cleavage of ERVWE2 and presentation to MHC II, we also investigated for putative cleavage sites nearby the shared epitopes of ERVWE2 and MOG. The analysis revealed several sites with high probability to serve as proteasomic cleavage sites (35 putative regions in MOG and 87 in HERV-W). No putative cleavage site was found nearby the region that matches to MPB.

## Discussion

### ERVEW2 analysis

Previous reports in this field focused on detection and characterization of the HERV-W transcripts found in MS patients (Garson et al., 1998; Blond et al., 1999a; Laufer et al., 2009; Roebke et al., 2010; Perron et al., 2012). In fact, the HERV-W Env detection in serum and brain of MS patients but not in controls sustains the association of retroviral activation and the disease (Perron et al., 2012). MSRV envelope transcripts come from different chromosomal loci, but most of them are related to the Xq22.3 chromosome (ERVWE2) (Laufer et al., 2009), which lack the 5′ LTR, gag, and part of the polymerase. This locus cannot account for retroviral particles associated with RT activity and MSRV subtype RNA as described (Perron et al., 1997; Garson et al., 1998; Komurian-Pradel et al., 1999), unless alternative upstream promoter regions exist, or the expression is 3′ LTR-mediated.

Although the putative promoter regions need to be validated in human samples, the presence of seven TFBs including Sp1 and AP-2 upstream the start codon of reference sequence identified in this study could explain the high level of transcripts reported despite the lack of 5′ LTR. Receptors for Sp1 protein are present in several eukaryotic genes involved with cellular differentiation and also in the LTRs of exogenous and endogenous retroviruses (Urnovitz and Murphy, 1996). Also, both Sp1 and AP-2 participate in transcriptional activation or repression of HERV-W genes in a tissue-dependent manner (Schön et al., 2001; Lee et al., 2003; Prudhomme et al., 2004).

Nevertheless, the fact that the ERVWE2 could be transcribed via alternative promoters does not mean that a functional protein is produced since the ERVWE2 DNA is N-terminally truncated. Intriguingly, some transcripts previously associated to this locus harbored a tryptophan (TGG) at site 39 instead (Laufer et al., 2009), suggesting that genetic polymorphism could allow the transcription of a complete ORF in MS patients.

This is not the case however. By sequencing the ERVWE2 locus of 84 individuals, including nine MS patients with HERV-W envelope activity detected in PMBC, we showed no evidence of genetic polymorphism that could revert the stop codon in any individual. Also, although our experiment to find retroviral mRNA was not designed to discriminate the source of transcripts, the slight Tm deviation among individuals may indicate that transcripts came from distinct sources. Taken together, our data support that retroviral transactivation may occur in distinct chromosomal loci, and despite no 5′ LTR and the incapability to generate a functional protein, ERVWE2 would be capable to be transcribed via alternative promoters. Nevertheless, experimental analyses of eventual alternative promoters will also be required before final conclusions may be drawn about their accuracy.

The reported detection of transcripts closely associated to this chromosome but harboring a tryptophan at codon 39 suggests two possible and non-exclusive explanations: (1) These mRNA are recombinant products generated *in vitro* (Laufer et al., 2009); and (2) The ERVWE2-associated mRNA were generated by an unfixed provirus, which have been formed earlier in human evolution and would then be represented by MSRV subtype sequences, as associated with extracellular retroviral particles and reverse-transcriptase (Dolei and Perron, 2009), In keeping with this, the subfamily 3 (that includes syncytin and ERVWE2) comprises most of the HERV-W members by which the intense activity extends to the last 5 million years (Costas, 2002). Thus, if new elements were generated after this period (i.e., after the origin of the genus *Homo*) (Jones et al., 1994) they may not have been fixed in the human genome and are present in the population as polymorphic insertions (Macfarlane and Simmonds, 2004).

### HERV-W and molecular mimicry

Usually, the immune system is tolerant to self-antigens, but in autoimmune disease the tolerance is abrogated to these antigens, leading to direct tissue damage mediated by immune response. The reasons that lead to this autoimmunity are still unknown, but viruses are good candidates to trigger this process.

The mechanisms by which viruses could induce autoimmunity include the expression of superantigens and molecular mimicry, whereas auto-reactive T cells are induced by epitopes shared between pathogen and host (self-antigens) (Miller et al., 2001). In this context, HERVs might act as both: encoding superantigens thereby result in enhanced inflammatory responses or mimicking self-antigens (Clausen, 2003; Rolland et al., 2006; Ogasawara et al., 2010).

As demonstrated before and confirmed here by in vitro and in silico analysis, several HERV-W envelope, including ERVWE2 are N-terminally truncated and do not produce a functional protein. However, since the transcription of these proviruses was exhaustively demonstrated, it is possible that simply expression and formation of truncated proteins is sufficient to elicit an immune response.

Alternatively, truncated ERVWE loci could skip the stop codons by generating spliced mRNAs. Although little information about HERV-W splicing is available (Blond et al., 1999b), HERV-H spliced mRNA can be found almost exclusively in MS patients (Christensen et al., 2003). There are also evidences of a pathogenic potential of spliced syncytin-1, as demonstrated by the presence of spliced mRNA in biopsies of testicular seminomas (Trejbalová et al., 2011). Despite no current evidence of spliced ERVWE mRNA in MS, one could hypothesize that cell-specific splicing factors might be involved in ERVW alternative splicing in brain cells, allowing the synthesis of ERVWE2 mRNAs without the stop codon region. Nevertheless, we found no evidence of donor and acceptor splicing sites in the ERVWE2 locus and its putative promoter region. Since the promoter regions as well as myelin and retroviral envelope similarity were predicted by in silico analyses only, further and dedicated studies are now needed to better characterize the possible involvement between HERV-W transcription and induction of autoimmunity.

In conclusion, the involvement of HERV-W envelope to the cross immunity in MS involves other element(s) than the ERVWE2 locus expression itself. As demonstrated here, the similarity between MOG and envelope is not restricted to the ERVWE2 locus, but it is extended to several HERV-W envelope genes from subfamily 3. It does imply that any HERV-W envelope from MRSV subfamily, together to other genetic or environmental co-factors, could trigger the immune system leading to a cross reaction against MOG. Additionally, the high heterogeneity among individuals and previous exposition to other viral antigens may have some impact on the transactivation of HERV-W proviruses and interfere with the activation of inflammatory cytokines.

## Concluding remarks

From our present observations, molecular mimicry is likely to provide an important avenue of research on these HERV proteins, which could turn to explain why the immune system targets specific self-proteins in certain autoimmune diseases. Since HERVs may be the molecular link between host's genetic factors and autoimmune diseases there is now a need for in-depth analysis of the active loci in the human genome to better clarify the role of these retroelements to the autoimmunity. Alternatively, if an unfixed active element related to the HERV-W family or specific spliced mRNA are indeed related to MS pathogenesis, very different methodological approaches must be considered for the purpose of their molecular isolation in selected productive cells from patients with MS.

### Conflict of interest statement

The authors declare that the research was conducted in the absence of any commercial or financial relationships that could be construed as a potential conflict of interest.
