# Erratum: Genomic analysis of ERVWE2 locus in patients with Multiple sclerosis: absence of genetic association but potential role of Human Endogenous retrovirus type W elements in molecular mimicry with myelin antigen

**DOI:** 10.3389/fmicb.2013.00345

**Published:** 2013-11-18

**Authors:** Camila Malta Romano

**Affiliations:** Departamento de Moléstias Infecciosas e Parasitárias – (LIMHC), Instituto de Medicina Tropical de São Paulo e Faculdade de Medicina, Universidade de São PauloSão Paulo, Brazil

**Keywords:** endogenous retroviruses, multiple sclerosis, MOG, immunopathogeny, genetic association, ERVWE2

The correct sequences of the primers described in this work are HW_MSchX_F-TGGGTGAAGTAAGTCCAACAG and HW_MSchX_R-TGAAGAACGTATCCAGCCTACA. The amplified fragment corresponds to nucleotides 21227–22122 of the human chromosome Xq22.3 (GenBank ID AL390039.10).

